# Fully Automated Fluorescent *in situ* Hybridization (FISH) Staining and Digital Analysis of *HER2* in Breast Cancer: A Validation Study

**DOI:** 10.1371/journal.pone.0123201

**Published:** 2015-04-06

**Authors:** Elise M. J. van der Logt, Deborah A. J. Kuperus, Jan W. van Setten, Marius C. van den Heuvel, James. E. Boers, Ed Schuuring, Robby E. Kibbelaar

**Affiliations:** 1 Department of Pathology, Pathology Friesland, Leeuwarden, The Netherlands; 2 Department of Pathology and Medical Biology, University of Groningen, University Medical Center Groningen, Groningen, The Netherlands; 3 Department of Pathology, Isala Klinieken, Zwolle, The Netherlands; University Medical Centre Utrecht, NETHERLANDS

## Abstract

HER2 assessment is routinely used to select patients with invasive breast cancer that might benefit from HER2-targeted therapy. The aim of this study was to validate a fully automated *in situ* hybridization (ISH) procedure that combines the automated Leica *HER2* fluorescent ISH system for Bond with supervised automated analysis with the Visia imaging D-Sight digital imaging platform. *HER2* assessment was performed on 328 formalin-fixed/paraffin-embedded invasive breast cancer tumors on tissue microarrays (TMA) and 100 (50 selected IHC 2+ and 50 random IHC scores) full-sized slides of resections/biopsies obtained for diagnostic purposes previously. For digital analysis slides were pre-screened at 20x and 100x magnification for all fluorescent signals and supervised-automated scoring was performed on at least two pictures (in total at least 20 nuclei were counted) with the D-Sight HER2 FISH analysis module by two observers independently. Results were compared to data obtained previously with the manual Abbott FISH test. The overall agreement with Abbott FISH data among TMA samples and 50 selected IHC 2+ cases was 98.8% (κ = 0.94) and 93.8% (κ = 0.88), respectively. The results of 50 additionally tested unselected IHC cases were concordant with previously obtained IHC and/or FISH data. The combination of the Leica FISH system with the D-Sight digital imaging platform is a feasible method for *HER2* assessment in routine clinical practice for patients with invasive breast cancer.

## Introduction

Breast cancer is the most common form of cancer in women with an incidence of 464,000 cases in Europe in 2012 [1]. The development and progression of breast cancer is influenced by the human epidermal growth factor receptor-2 (HER2) [2–3]. Specifically, amplification of the *HER2* gene (*ERBB2*) is found in approximately 10% to 20% of invasive breast carcinoma [4–5] and results in the overexpression of HER2 protein and is associated with a poor prognosis [6–8]. Because of the high incidence of HER2 overexpression, HER2 was considered a candidate for targeted therapy. Treatment with the monoclonal antibody trastuzumab inhibits the proliferation of tumor cells that overexpress HER2 [9–11] and thereby significantly increases the survival of HER2-positive patients [12–13]. Prediction of HER2-directed therapy is determined by assessing HER2 status with immunohistochemistry (IHC) and reflex testing of IHC++ with *in situ* hybridization (ISH) techniques [4–5]. The successful implementation of HER2 testing in medical practice is well established. All women who received trastuzumab had a HER2 test, and more than 95% of these women had a positive HER2 status in a cohort of lymph node-positive breast cancer or 2+ cm tumors in size diagnosed between 1999 and 2007 enrolled at eight Cancer Research Network study sites across the United States [14]. However, the inaccuracy of HER2 testing remains a major issue. Approximately 10–20% of HER2 tests performed in local laboratories could not be confirmed by a central laboratory [15, 16]. In addition, in about 40% of breast cancer patients with a HER2 borderline test result, the clinical practice guidelines were not followed, in that reflex FISH testing was not offered to these patients to clarify HER2 status [14]. As a consequence, patients with a false-positive test or a fraction of patients with only a borderline IHC test are treated at high costs and potential cardiotoxicity without having benefit from their therapy.

In most laboratories *HER2* ISH testing remains a time-consuming and technically challenging manual molecular diagnostic assay because of the risk for human processing errors and subjective interpretation. The American Society of Clinical Oncology (ASCO) published a guideline for HER2 testing with recommendations to improve the standardization of the assay by the automation of the total procedure, from staining up to analysis [15, 17]. Recently, this has been partly achieved by the development of fully automated staining procedures for both fluorescent- [18] and bright-field ISH [19–20]. However, the standardization of *HER2* gene status testing could be further improved by automating the analysis of the slides. This will introduce a more objective classification of *HER2* gene status. The FISH analysis systems described by Netten *et al*., CytoFISH, FishJ and MetaSystems have shown the ability to standardize *HER2* analysis by automatic counting of fluorescent signals [21–24]. These digital analysis systems are based on automated nucleus selection, followed by automated spot counting and optionally human corrections can be made. Automated analysis was highly concordant with manual analysis [23–24]. Recently, Visia imaging has developed the D-Sight digital imaging platform which integrates both the generation of digital images and automated image analysis.

The objective of this study was to validate the standardized detection of *HER2* gene status using a fully automated ISH procedure, combining the automated Leica *HER2* fluorescent *in situ* hybridization (FISH) staining system for Bond and subsequent supervised automated analysis with the Visia imaging D-Sight digital imaging platform. Validation was achieved by comparing these data with data obtained previously by manual analysis of DAKO IHC, Abbott FISH using tissue microarray (TMA; n = 328) and full-sized slides (n = 100) from formalin-fixed paraffin-embedded (FFPE) tissue specimens of invasive breast cancer.

## Materials and Methods

### Tissue specimens

The validation process was divided in three phases and in total formalin-fixed paraffin-embedded (FFPE) tissue specimens of 448 invasive breast cancer cases were analyzed (see Table 1). Firstly, the use of automated Leica FISH staining and the suitability of the settings of the D-Sight *HER2* FISH analysis module for digital analysis was tested on 20 full-sized tissue slides. Secondly, technical validation was set up on a larger series of cases using Tissue Micro Arrays (TMAs). For TMA design paraffin blocks from the University Medical Center Groningen (UMCG) and Isala Klinieken Zwolle were used as reported by Dekker *et al*. [25]. From each tumor three cores with a diameter of 0.6 mm were collected. Thirdly, validation on clinical routine specimens was performed on 100 resections/full-sized slides or biopsies (18%) consecutively collected at Pathology Friesland (divided in 50 selected DAKO IHC 2+ cases and 50 unselected cases with different IHC scores. The tissue blocks that were used in this study were all collected during routine diagnostics. According to Dutch law, these can be freely used after anonymizing the tissues, provided these are handled according to national ethical guidelines (‘Code for Proper Secondary Use of Human Tissue’, Dutch Federation of Medical Scientific Societies).

**Table 1:** Characteristics tested tissue specimens of patients with invasive breast cancer with Leica *HER2* FISH.

**Validation**	**N**	**Tissue specimen**	**Pathology lab**	**IHC score (DAKO)**	**Data ISH method**
Settings automated	20	Resection	Groningen 2007	5x 0	Abbott
staining and analysis				5x 1+	Abbott
				5x 2+	Abbott
				5x 3+	Abbott
Technical	328	Resection TMA	Groningen / Zwolle 2007	Variable	Abbott
Clinical	50	Resection / biopsy	Friesland 2011	Selected 2+	Abbott
	50	Resection / biopsy	Friesland 2012 (consecutive)	Unselected (0–3+)	2+ Abbott

IHC, immunohistochemistry; TMA, tissue micro array.

### Fully manual HER2 testing using the DAKO HercepTest and Abbott *HER2* FISH test

As reference method we used a fully manual HER2 testing procedure (both staining and analysis). Firstly, immunohistochemistry (IHC) for HER2 was performed manually with the DAKO HercepTest (Glostrup, Denmark) at Pathology Friesland, as described previously [26]. Secondly, *HER2* gene status was determined for TMA and IHC 2+ cases using a conventional manual procedure with the PathVysion *HER2* DNA Probe kit, (Abbott Molecular, Illinois, United States) performed in the routine ISO15189-certified laboratory of Molecular Pathology in the UMCG (referred to as Abbott FISH). After dewaxing, unmasking of the target nucleic acids at 120°C for 7 min in TRIS/EDTA pH9.0 buffer in pressure cooker, pretreatment with RNase at 37°C for 10 min and with pepsin at 37°C for 1 hr, denaturation and hybridization were performed according the manufacturer´s instructions. Data on Abbott FISH will be reported separately [27].

Manual FISH analysis started by scanning of the slide/core for areas of amplification. In full-sized slides with IHC 2+ scores a pathologist marked the area of interest. Scoring for *in situ* hybridization was performed by two independent observers according to the American Society of Clinical Oncology (ASCO) guidelines 2007 [15]. Discordant cases were scored by a third observer. The *HER2* and chromosome 17 (*CEP17*) signals were counted in at least 20 non-overlapping nuclei and the ratio between *HER2* and *CEP17* was calculated. When the ratio was < 1.8, the *HER2* gene status was defined as non-amplified, while a ratio ≥2.2 was considered as *HER2* gene amplification. Ratios between 1.8 and 2.2 were counted as equivocal and an additional number of 20 nuclei were scored. When this yielded a ratio <2.0, the *HER2* gene status was defined as non-amplified and a ratio ≥2.0 was classified as *HER2* gene amplified.

### Fully automated Leica *HER2* FISH staining and supervised automated analysis

In this study, we investigated the performance of a fully automated *HER2* gene testing procedure (both staining and analysis; referred to as Leica FISH). Fully automated FISH was performed on the Leica Bond slide-staining system using the FISH *HER2* staining kit (TA9217) from Leica Microsystems (Newcastle, UK) according to manufacturer’s instructions with exception of adjustments to optimize pretreatment conditions. It is a fluorescent *in situ* hybridization product and contains PathVysion + LSI + *HER2/CEP17* FISH probes supplied by Abbott Molecular Inc. The LSI *HER2* probe is a 190 Kb SpectrumOrange directly labeled fluorescent DNA probe for the *HER2* locus (17q11.2-q12) and the *CEP17* DNA probe is a 5.4 Kb SpectrumGreen directly labeled fluorescent DNA probe specific for the alpha satellite DNA sequence at the centromeric region of chromosome 17 (17p11.1-q11.1).

Breast cancer tissue specimens were routinely processed and sectioned into 4 μm slides. Before staining, slides were incubated at 60 °C for one hour and Enzyme 5 (i.e. proteinase K) was freshly prepared by dilution (1:300) of Enzyme Concentrate 2 of the Leica *HER2* FISH staining system in Enzyme diluent. All other components are ready-to-use. Prior and after use the staining system was stored at 2–8 °C. The default automated staining protocol was as follows: dewaxing, unmasking of the target nucleic acids at 97°C for 25 min (adjusted to 30 min in this study) with Bond epitope retrieval solution 1 (i.e. citrate buffer), pretreatment with Enzyme 5 at 37°C for 25 min (adjusted to 5 min in this study), denaturation at 95°C for 10 min, hybridization with probes at 37°C for 12h, stringency washing with Post hybridization wash solution 2 at 48°C for 4 min, dehydration (2x alcohol 96% and air-dried) and finally slides were mounted manually in vectashield with DAPI (1:1 diluted in vectashield) and sealed with colorless nail-polish. Stained sections were stored at -20 °C in the dark until evaluation to prevent fading of the fluorescent signals. Incubation times of heat induced epitope retrieval and enzyme treatment were adapted because default setting resulted in suboptimal staining of the slides with no/weak signals for analysis.

In this study, we evaluated the *HER2* gene status of 380 TMA cases for Leica FISH. Fifty-two TMA cases yielded incomplete ISH scores due to absence of tissue cores, folding of tissue cores, too low amounts of invasive breast cancer cells for scoring or because no FISH signals were detected (only three cases). Therefore, data of 328 TMA cases could be compared with Abbott FISH. These staining issues were not encountered with full-sized slides or biopsies (= clinical practice).

Analysis was performed by supervised automated scoring with the Visia imaging D-Sight digital imaging platform (exclusively distributed in Europe by Menarini Benelux). At first, slides were scanned at 4x magnification to obtain an overview of the tissue specimen. In case of slides with IHC 2+ scores the marked area of interest (invasive tumor identified by a pathologist) was visible. To account for possible heterogeneity, slides were pre-screened at 20x magnification in the SpectrumOrange channel setting and checked for *HER2* gene amplification. This was repeated at 100x magnification on a selected area of interest and all fluorescent signals were checked for signal intensity using the SpectrumOrange, SpectrumGreen and DAPI filter channels. At least 4 areas of interest were selected for analysis and images were taken automatically by the monochrome camera for all fluorescent signals using Z-stack acquisition (13 stacks/planes, distance 0.5 μm). Captured images from different focus planes were combined generating a picture with all signals clearly detectable after removal of unspecific background.

**Fig 1:**
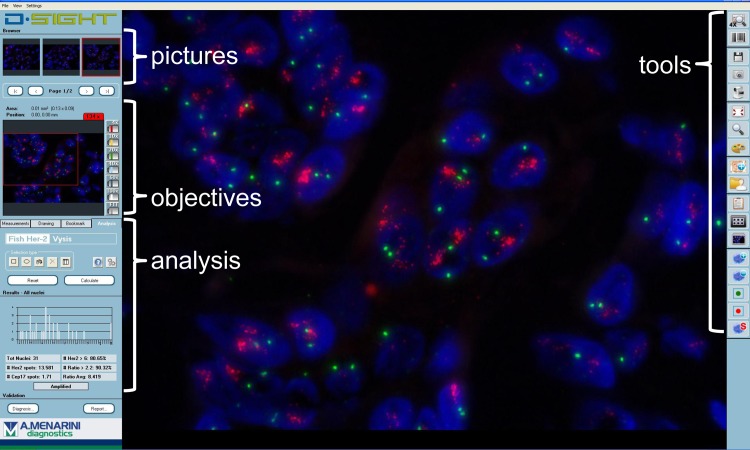
The design of the D-Sight *HER2* FISH analysis module (software version 2012 2.1.2). “Pictures”: at least 4 areas of interest are selected for analysis and pictures are taken automatically. Captured images from different focus planes are combined generating a picture with all signals clearly detectable after removal of unspecific background. “Objectives and tools”: automated nucleus selection and spot counting of the red (*HER2*) and the green (chromosoom 17 = *CEP17*) signal is evaluated and corrected by the observer if needed. “Analysis”: supervised-automated scoring is performed on at least two pictures and the ratio between *HER2* and *CEP17* was generated.

Supervised automated scoring was performed on at least two pictures (in total at least 20 nuclei were counted) with the D-Sight *HER2* FISH analysis module (software version 2011 1.3.15; [28]) and the ratio between *HER2* and *CEP17* was generated. Automated nucleus selection and spot counting of the red (*HER2*) and the green (*CEP17*) signal was evaluated and corrected by the observer if needed. Nuclei without red and green signals were automatically excluded during the nucleus selection step by the image analysis software. The observers need to indicate the areas of interest in the tissue on the slide before the D-Sight analysis module will count *HER2* and *CEP17* copies per selected area automatically. Heterogeneity will be observed by analysis of the data by the observers (= supervised automated scoring). Fig 1 shows an example of the latest software version (2012 2.1.2) of the D-Sight *HER2* FISH analysis module.

Scanning and scoring was conducted by two independent observers and upon discordance a third analysis was made by the two observers together to obtain a final (consensus) score. The quality of the Leica FISH was monitored by inclusion of an internal control in each run and consisted of a TMA of earlier analyzed invasive breast cancer tumors with different expression of HER2.

The *HER2* gene status determined by the available Visia imaging D-Sight *HER2* FISH analysis module (software version 2011 1.3.15) is based on the FISH scoring criteria according to ASCO guideline 2007 [15] as described in the previous section. A new software version (2.5.0) based on the recently updated ASCO guideline [17] will be available in December 2014 (personal communication with Menarini Benelux). This means that the data in the present study are based on the 2007 ASCO guideline and cases with *HER2*/CEP17 ratio of <2.0 but *HER2* copy number of ≥6.0 are considered *HER2*-negative, while in the updated guideline such cases should be considered *HER2*-positive. To investigate whether the new guideline would affect the interpretation of the *HER2* gene status of our validation cohort, we recalculated the *HER2* gene status of the 328 TMA cases stained with Abbott FISH, and found that in total 9 cases (2.7%) had a different interpretation. All 9 cases involved an equivocal result, showing changes from equivocal ratio <2.0 to negative, equivocal ratio ≥2.0 to positive and *vice versa*. Most importantly, all these changes had no consequence for the therapy choice of the patients using the criteria of either the old or new guideline. Therefore we assume that the new ASCO guideline will not affect the overall conclusions in this manuscript.

### Statistics

Concordance rates between different combinations of staining and analysis methods were determined and Cohen’s κ statistics was performed using GraphPad QuickCals software.

## Results

At first, the automated Leica FISH staining and settings of the D-Sight *HER2* FISH analysis module for digital analysis (fully automated ISH procedure) were evaluated and showed an 95% (κ = 0.94) agreement with the original Abbott FISH analysis. Only one discordant score was found and depicted as an equivocal (< 2.0) ratio with Leica FISH versus a non-amplified ratio with Abbott FISH (see 3x3 analysis in Table 2). However, the final interpretation was the same, because the *HER2* gene status was not amplified for both ISH procedures (see 2x2 analysis in Table 2). We concluded that the automated Leica FISH staining followed by supervised-automated digital analysis was suitable for further evaluation.

**Table 2:** Comparisons of automated Leica *HER2* FISH with digital analysis and manual Abbott *HER2* FISH with manual analysis in 20 full-sized slides of tissue specimens used for TMA blocks of invasive breast cancer.

			Abbott FISH (manual procedure)		
			Amplified^a^	Equivocal^b^	Non-amplified^c^
**Leica FISH**		Amplified	6	0	0
**(automated procedure)**		Equivocal	0	1 (≥2.0)^d^	1 (<2.0) ^d^
		Non-amplified	0	0	12
**Overall agreement**	3x3^e^	95.0% (κ = 0.94 (weighted))			
	2x2^f^	100%			

FISH, fluorescence *in situ* hybridization.

^a^ ratio ≥ 2.2.

^b^ ratio = 1.8–2.2.

^c^ ratio <1.8.

^d^ between brackets equivocal ratio after additional count of 20 nuclei <2.0 or ≥2.0.

^e^ 3 categories (amplified / equivocal / non-amplified).

^f^ 2 categories (amplified ratio ≥ 2.0 / non-amplified ratio <2.0).

Secondly, the feasibility of using the D-Sight digital imaging platform for supervised automated analysis of the automated Leica *HER2* FISH staining (fully automated ISH procedure) was validated by comparison to the original Abbott FISH data (manual ISH procedure). In 35 TMA cases an amplification of *HER2* (ratio ≥ 2.0) was identified by Leica FISH compared to 38 TMA cases by Abbott FISH. Overall, the agreement of Leica FISH with Abbott FISH was 98.8% (κ = 0.94; see Table 3).

**Table 3:** Comparisons of automated Leica *HER2* FISH with digital analysis and manual Abbott *HER2* FISH with manual analysis in tissue micro arrays (TMAs) including 328 invasive breast cancer tissue specimens.

			Abbott FISH (manual)		
			Amplified^a^	Equivocal^b^	Non-amplified^c^
**Leica FISH (automated)**		Amplified	34	1 (1x ≥2.0)^d^	0
		Equivocal	2 (≥2.0) ^d^	1 (1x <2.0) ^d^	4 (**1x** ≥**2.0**; 3x <2.0)^d,g^
		Non-amplified	**2** ^**g**^	4 (**1x** ≥**2.0**; 3x <2.0)^d,g^	280
**Overall agreement**	3x3^e^	96.0% (κ = 0.89 (weighted))			
	2x2^f^	98.8% (κ = 0.94)			

FISH, fluorescence *in situ* hybridization.

^a^ ratio ≥ 2.2.

^b^ ratio = 1.8–2.2.

^c^ ratio <1.8.

^d^ between brackets equivocal ratio after additional count of 20 nuclei <2.0 or ≥2.0.

^e^ 3 categories (amplified / equivocal / non-amplified).

^f^ 2 categories (amplified ratio ≥ 2.0 / non-amplified ratio <2.0).

^g^ Bold characters indicate discordant results.

**Fig 2:**
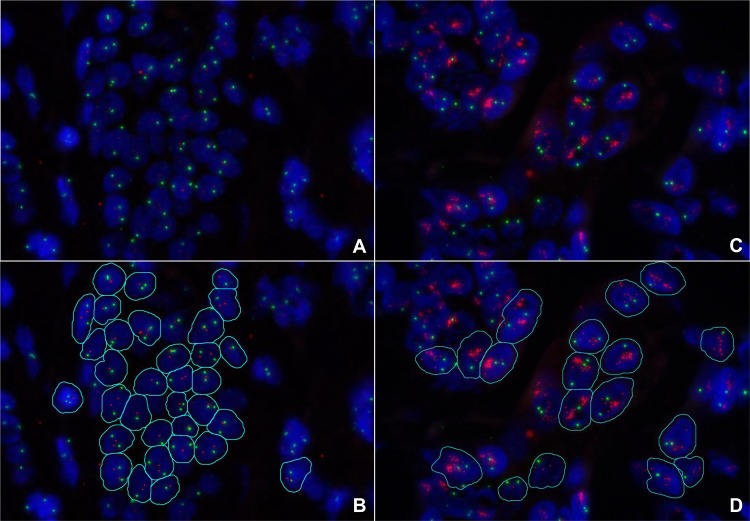
The appearance of Leica FISH *HER2* staining. Representative pictures of HER2 non-amplified invasive breast cancer specimens are shown in A) with a *HER2* (red signals) to chromosoom 17 (= *CEP17*; green signals) ratio <2 and C) with supervised-automated nuclei and spot detection. Examples of *HER2* amplified invasive breast cancer specimens are depicted in B) with a *HER2*/*CEP17* ratio >2 and D) with supervised-automated nucleus and spot detection. DAPI counterstaining and original magnification with 100x objective.

Representative pictures of Leica FISH for non-amplified and amplified *HER2* gene status are depicted in Fig 2, with or without supervised-automated nucleus and spot detection.

Staining of Abbott FISH and Leica FISH was repeated on 3 of the 4 discordant cases using full-sized sections of the original tumor tissue. For one case no material was left. In 2 of 3 retested cases Leica FISH was concordant to Abbott FISH (amplified *HER2* gene). Apparently, the selected area with amplified *HER2* gene on full-sized slides was not present on the TMA slide stained with Leica FISH in these 2 cases. Probably sampling errors occurred in these cases when TMA were made. The other discrepant case remained discordant between Leica (equivocal ratio <2.0) and Abbott (equivocal ratio >2.0) FISH analysis.

Discrepant cases involve ISH with equivocal scores in 2 of 4 cases. Therefore, results of these four cases were also compared to 5 other ISH staining methods as reported recently [27]. The consensus between the different ISH methods performed on these cases is low, maximally 4/7 scores were concordant per case. This indicates that cases with equivocal scores are difficult cases for *HER2* gene assessment.

Overall agreement between Leica FISH and Abbott FISH was very high after revision 99.4% (κ = 0.97).

Finally, the use of the automated Leica *HER2* FISH staining in combination with automated analysis for assessment of the *HER2* gene status in FFPET invasive breast cancer specimens in routine clinical practice was validated on 100 resections/full-sized slides or biopsies. Results were compared to DAKO IHC scoring and Abbott FISH data for IHC 2+ tumors. The initial Leica FISH failure rate (no fluorescent signals were detected) was 3% (3/100). Repeated testing of all failed Leica FISH samples was successful when adjusting the incubation time for enzyme pretreatment from 5 min to 1 or 2 min.

The 100 slides with resection or biopsy tissue were divided into two groups. Firstly, the *HER2* gene status was evaluated in 50 selected DAKO IHC 2+ cases. The overall agreement of Leica FISH with Abbott FISH was 93.8% (κ = 0.88; see Table 4). Three tumors were non-amplified with Leica FISH, but amplified with Abbott FISH. These cases were considered to be discordant and reanalysis of Leica FISH confirmed amplification in 2/3 tumors. This experience prompted us to adjust the procedure for supervised-automated digital analysis in such way that observers should perform a pre-screen of the tissue specimens at 20x magnification followed by one at 100x magnification. Application of this workflow on the 50 consecutively collected full-sized slides did not yield any discrepancies. The remaining discrepant case was stained again with Abbott FISH and yielded a non-amplified score similar to Leica FISH, but discordant to the initial result of Abbott FISH. This could be clarified by the fact that the slide of the first Abbott FISH staining contained 3 biopsies, whereas the biopsy in the middle was missing in the new Abbott FISH and Leica FISH staining. This case involves a tumor with heterogeneity for *HER2* gene status. The biopsy in the middle showed amplified *HER2* gene with Abbott FISH but the two other biopsies did not.

**Table 4:** Comparisons of automated Leica *HER2* FISH with digital analysis and manual Abbott *HER2* FISH with manual analysis of 50 invasive breast cancer tissue specimens (resection / biopsy) with IHC 2+ scores.

			Abbott FISH (manual)		
			Amplified^a^	Equivocal^b^	Non-amplified^c^
**Leica FISH (automated)**		Amplified	21	0	0
		Equivocal	0	0	0
		Non-amplified	**3** ^**e**^	0	24
**Overall agreement**	3x3^d^	93.8% (κ = 0.88 (weighted))			

FISH, fluorescence *in situ* hybridization.

^a^ ratio ≥ 2.2.

^b^ ratio = 1.8–2.2.

^c^ ratio <1.8.

^d^ 3 categories (amplified / equivocal / non-amplified).

^e^ 2x) reanalysis Leica FISH confirms amplification Abbott FISH; 1x) new staining Abbott FISH confirms non-amplified score Leica FISH (first Abbott FISH contained 3 biopsies, whereas the biopsy in the middle is missing in the new Abbott FISH and Leica FISH). 2x missing data because no signals were detected with Leica FISH.

Secondly, the Leica FISH analyses of 50 unselected tumors with different IHC scores were all in agreement with DAKO IHC scores and Abbott FISH data for IHC 2+ tumors (see Table 5).

**Table 5:** Comparisons of Leica *HER2* FISH and DAKO HER2 IHC of 50 consecutively collected invasive breast cancer tissue specimens (resection / biopsy) from routine practice.

			DAKO HER2 IHC			
			0	1+	2+	3+
**Leica *HER2* FISH**		Amplified^a^	0	0	2	8
		Equivocal^b^	0	0	0	0
		Non-amplified^c^	21	17	1	0

IHC, immunohistochemistry; FISH, fluorescence *in situ* hybridization. 1x missing data because no signals were detected with Leica FISH.

^a^ ratio ≥ 2.2.

^b^ ratio = 1.8–2.2.

^c^ ratio <1.8.

## Discussion

Recently, more standardized *HER2* gene testing has been achieved by the development of fully automated ISH staining systems and high concordance rates were found in comparison with manual ISH staining tests [18–20]. Standardization could further be improved by using objective scoring systems on digital FISH images developed on different platforms [24, 29]. However, so far the combination of automated staining with automated digital analysis of invasive breast cancer tumors has not been studied yet.

In order to investigate whether the reliability *HER2* ISH testing can be improved, we compared automated Leica *HER2* FISH staining followed by supervised automated digital analysis with manual Abbott FISH staining and subsequent manual analysis aimed at the use in daily practice of a routine histopathology laboratory. We found a very high concordance (98.9%, κ = 0.94) on 328 TMA samples and 93.8% (κ = 0.88) on 50 selected IHC 2+ cases (resections or biopsies). A similar performance was found in a recent study by Öhlschlegel *et al*. [18], comparing automated Leica *HER2* FISH staining and subsequent manual analysis and manual Abbott *HER2* FISH staining followed by manual analysis. The authors reported an overall concordance rate of 96% (κ = 0.92) and 93.3% for IHC 2+ breast cancer biopsies. These and our results stated the feasibility of automated FISH *HER2* staining.

Our performance of supervised automated digital analysis (of automated stained ISH slides) was comparable with other digital analysis systems that included automated nucleus selection (on manually stained ISH slides). Theodosiou *et al*. [30] found an overall concordance of digital analysis with manual analysis of 92.8% among 100 breast cancer cases. This was in agreement with data reported by Furrer *et al*. [24]. Concordance was 100% for non-amplified cases and 96.9% (100% after human correction) for amplified cases. Notably, both studies recommended to exclude equivocal cases, because of the suboptimal performance they encountered when applying digital analysis on these cases. We performed digital analysis without selection of FISH *HER2* gene status, the analyses were robust and fully in concordance with the previous manual analyses. We encountered a few difficult cases with discordant results which, in agreement with the findings mentioned above, almost all were found in equivocal cases. The discrepancies can be caused by different staining methods, inherent biological properties, the analysis method or combinations of these.

Advantages for clinical practice of this automated standardized *HER2* gene testing are: 1) further standardization of the total procedure, 2) reducing human mistakes and 3) yields archived data, which are accessible for review and objective classification. In our experience, turnaround time and hands on time are reduced by automated testing. However, these factors were not quantified and may by subject of future investigations. Disadvantages of FISH techniques are fading of fluorescent signals, hence in time the diagnostic data are not accessible anymore, and secondly it is not easy, sometimes impossible, to review the exact same area on the slide of a case that was evaluated manually by one of the observers before. Using (automated) digital analysis the original diagnostic images (= coordinates) are archived and accessible for review. By using web-based techniques images can easily be reviewed simultaneously at different places. In terms of transparency and quality control these are pivotal features.

As mentioned above, an advantage of supervised automated analysis is a more objective classification of the FISH staining compared to manual analysis, because the nucleus selection and spot counting in the areas of interest is automated and not depending on the subjectivity of the observer. Choosing the area of interest to our opinion is not another level of subjectivity introduced by supervised analysis but is inherent to both manual and automated methods. Because areas of heterogeneity might be present fully automated image analysis and scoring is not possible. The system is not able to identify these areas and therefore areas of interest should be indicated and the proposed nucleus selection confirmed by the observer. After this, automated analysis will calculate *HER2/CEP17* ratio’s for each area. This indicates that experienced observers are needed for reliable scoring of *HER2* gene status. For this reason we prefer to designate the procedure as “supervised automated scoring”.

We did not evaluate the cost-effectiveness in this study. In the future, important issues to take into consideration in this matter are that automated staining kits are more expensive than reagents used for manual staining. Hands on time and therefore expenses for technicians to perform FISH staining is much higher in case of a manual method. A digital analysis systems is more expensive than a conventional microscope, but automated analysis will generate a more objective classification compared to manual analysis. Automated analysis takes longer than manual analysis, however digital images can be archived and are accessible for review and this is not the case with manual analysis.

The reported method for HER2 testing is restricted to FISH analysis and does not include automated analysis of HER2 IHC slides. Further improvements to the process of HER2 testing could therefore be made by including automated HER2 membrane analysis of IHC slides. The D-Sight digital imaging platform actually has the ability to match IHC with FISH slides and is facilitated with a HER2 IHC membrane analysis module. Validation of this analysis method would be an opportunity to further optimize the HER2 testing procedure in the future.

Summarizing, our fully automated ISH procedure showed a similar performance as found with a fully manual ISH procedure (> 95% concordance rates). Therefore, standardization of the total procedure of *HER2* ISH testing could be achieved by adding supervised automated digital analysis to automated staining. These results meet the criteria for test validation as stated by ASCO [15]. The reported method, based on supervised automated analysis of digital images, is feasible for the assessment of *HER2* gene status in routine clinical practice for patients with invasive breast cancer and yields images of high quality which are subsequently accessible for review and transparent reporting.
